# Mapping and characterization of a quantitative trait locus resistance to the brown planthopper in the rice variety IR64

**DOI:** 10.1186/s41065-019-0098-4

**Published:** 2019-06-26

**Authors:** Meng Yang, Ling Cheng, Liuhui Yan, Wan Shu, Xinyi Wang, Yongfu Qiu

**Affiliations:** 1 State Key Laboratory for Conservation and Utilization of Subtropical Agro-bioresources, Agricultural College, Guangxi University, Nanning, 530005 China; 2 Maize Research Institute, Guangxi Academy of Agricultural Sciences, Nanning, 530007 China; 3 College of Agriculture, Yangtze University, Jingzhou, 434025 China

**Keywords:** Brown planthopper (*Nilaparvata lugens* Stål), Tolerance, Genetic mapping, Preliminary-near-isogenic line (pre-NIL), Insect resistance mechanism

## Abstract

**Background:**

Rice planthoppers (main brown planthopper, *Nilaparvata lugens* Stål; BPH) was one of substantial threats to Asia rice production as its serious destruction and difficulties in control under field conditions. Notably, host-plant resistance was proved to be one of the effective ways to manage the pest. And stronger virulence will probably emergence when continuous use of insecticides. Therefore, more resistance genes with different resistance mechanisms were needed to be detected and then applied in the rice breeding practice.

**Results:**

Resistance genes in the rice variety IR64 were evaluated considering the seedling bulk test and seedling survival rate. As a result, a locus with a large LOD score of 7.23 was found between markers RM302 and YM35 on chromosome 1. The locus explained 36.9% of phenotypic variation and was tentatively denominated *Bph37*. Moreover, *Bph1* was detected to be harbored by the markers RM28366 and RM463, and had the largest LOD score of 2.08, explaining 7.7% of phenotypic variance in the same mapping population. Finally, the preliminary-near-isogenic-lines (pre-NILs) carrying *Bph37* exhibited significant tolerance to the insects. But no antibiotic or antixenotic effects were observed in the resistant plants when infested with the insects.

**Conclusions:**

We mapped one major BPH resistance gene *Bph37* in consideration of seedling survival rate and the resistance lines showed tolerance to BPH. The detected gene should be beneficial for understanding the resistance mechanism of rice to BPH and for insect-resistance rice breeding programs.

## Background

Insect pests represent a major constraint in global agriculture, reducing crop yield and quality. The brown planthopper (BPH), *Nilaparvata lugens* (Stål), one of the most devastating insect pests of rice (*Oryza sativa* L.), occurs widely in South, Southeast, and East Asia, as well as in the South Pacific islands and Australia. This insect uses its stylet to pierce the leaf sheath phloem sap and assimilate nutrients such as sucrose, amino acids, potassium, and ATP in the vascular bundle [14]. Simultaneously, it can transmit viruses, such as grassy stunt and ragged stunt, into the rice cultivars [19], and might be associated with serious diseases, leading to retardation of rice plant growth and flavescence on leaves [3, 38]. Heavy infestations can harm the rice plant, leading to complete drying and the occurrence of ‘hopper burn’. Application of chemical pesticide is a conventional and widely used method to control pests. However, this method increases the cultivation cost and kills the natural enemies. In addition, it will be easy to cause the BPHs outbreak [34]. Over the long term, the most economic and efficient way to control the insect is to identify BPH resistance genes in rice and to subsequently breed resistant varieties.

Plants have evolved various strategies to adapt to the external environment. Utilization of host-plant resistance genes in rice should be preferential measures for BPH management. To date, 36 major BPH resistance genes have been identified from cultivated varieties and wild rice species [23]. Varieties carrying the major resistance genes *Bph1*, *bph2*, or *bph4* have been widely used in countries of Southeast Asia [16]. However, the resistance of these varieties was lost after 2–3 years with the development of new BPH populations, biotypes 2 and 3 [9, 13, 25]. In addition, most of the major BPH resistance genes have been detected by the seedling bulk test and shown to confer an antibiotic effect on the insects. Therefore, it is difficult for the gene to play an important role in the durable resistance of the cultivated rice varieties. Overall, improving durable resistance to BPH in rice varieties remains challenging. Fortunately, pyramiding different resistance genes/QTLs is an effective way of increasing the level of resistance or improving the durability of resistance [1, 2, 27]. The rice variety IR64 presents moderate resistance to BPH and has been widely cultivated for more than 10 years in the rice cultivation areas of Southeast Asia [8, 10]. This variety carries one major resistance gene, *Bph1*, and several associated minor resistance QTLs [1, 2, 8]. This phenomenon was observed when pyramiding *Bph6* and one antixenosis QTL, *qBph8*(*t*) [28]. These examples suggest that the identification and characterization of different types of resistance genes/QTLs associated with BPH resistance is both important and imperative in practical rice breeding programs.

Generally, plants may employ antixenosis, antibiosis, or tolerance to insects with respect to physiological function [1, 18]. Previous research of IR64 has demonstrated each of these mechanisms with regard to BPH–rice interactions [1, 2, 8]. Subsequently, several major BPH resistance genes, including *Bph14*, *Bph6*, and *Bph9*, were reported to confer resistance via two different mechanisms [11, 26, 40]. However, relatively few studies of tolerance resistance genes/QTLs have been taken as a major gene/QTL for gene mapping and characterization. For example, *BPH7* was considered to confer tolerance to BPH insects after it was mapped by a seedling bulk test [29]. Recent research conducted by Du et al. [12] indicated that rice plants can escape drought through an ABA-dependent pathway. Therefore, it is essential to identify the tolerance or antixenosis genes/QTLs associated with BPH resistance and to study the mechanisms through which they act. It is beneficial to understand the different resistance mechanisms and to breed durable BPH resistance varieties.

Previous studies have shown that the rice variety IR64 has durable and medium resistance to BPH [1, 8]. Recent insect resistance tests also indicated that this rice variety has moderate resistance to the BPH population (mainly biotype 2) collected from a rice field at Nanning, Guangxi [7, 37]. Notably, IR64 carries one major resistance gene, *Bph1*, and several minor QTLs that confer antixenosis or tolerance to the BPH insects [8]. However, a rice variety containing *Bph1* became susceptible with the development of BPH biotype 2 [2, 8], which suggests that the other tolerance or antixenotic genes/QTLs play more important roles in resistance to BPH. Based on this point, we simultaneously evaluated the resistance level conferred by *Bph1* using the conventional seedling bulk test and surveyed the seedling survival rate associated with tolerance by F_2:3_ mapping population. As it is difficult to conduct a host choice test with the same population in the green house, antixenosis was not evaluated in the present study. As a result, one locus with a large LOD score was found between the markers RM302 and YM35 on chromosome 1. And *Bph1* was also detected in the same mapping population, which explained lower phenotypic variation.

## Materials and methods

### Plant materials and mapping population

The rice variety IR64 has been reported to contain one major BPH-resistance gene, *Bph1*, and other minor resistance QTLs associated with settling, oviposition, or tolerance [1, 33]. KWQZ, an *indica* rice line, was used as a susceptible parent for the crosses. Both were collected from Insititute of Chinese Crop Germplasm. An F_2:3_ mapping population consisting of 122 families derived from a KWQZ/IR64 cross was applied to identify and map the genes/QTLs.

To generate preliminary-near-isogenic-lines (pre-NILs) containing the target gene/QTL, the positive F_1_ hybrids were backcrossed with KWQZ twice and then self-pollinated once. Individuals of each generation were detected by tightly linked markers to obtain the positive plants. In this way, we obtained homozygous BC_2_F_2_ lines carrying one or two resistance genes/QTLs, which were used to analyze BPH resistance.

### BPH insects and evaluation of resistance

The BPH insects were collected from rice fields in 2013 in Nanning, China, and reared on TN1 (a susceptible *indica* variety) plants in a greenhouse at 26–30 °C at Guangxi University. A predominant biotype 2 was detected in most of the rice-growing regions in China [7, 37].

A seedling bulk test was performed on the F_2:3_ families as described by Qiu et al. [26] to map the resistance gene. One line of IR64, KWQZ, and TN1 each was taken as control and randomly sown among the tested lines. Seedlings were grown in a greenhouse under natural light at 26–30 °C. Each seedling will be treated with 2–3 instar nymphs at an average level of eight at the third-leaf stage (approximately 13 days after seeding). When all the TN1 seedlings died (scored as 9), each seedling was given a score of 0, 1, 3, 5, 7, or 9, as described by Huang et al. [15]. The experiments were conducted twice. The resistance score of each F_2_ individual was then inferred from the weighted average of the seedlings scores in the corresponding F_2:3_ families. The same methods were applied to evaluate the level of BPH resistance of the pre-NILs carrying one or two resistance genes/QTLs.

For detection of the tolerant genes/QTLs, the seeding method was identical to that used to test for resistance genes. The 3-week-old seedlings were treated with 2–3 instar BPH nymphs at an average level of five insects per seedling and covered with a fine light-transmitting mesh enclosure (58 × 38 × 9 cm). Each seedling would be evaluated when all the TN1 seedlings died (survival rate 0, about 20 days after infestation). Those that were green or presented activity were considered as surviving individuals; the surviving plants of each line were then counted. The survival rate of each F_2_ individual was then inferred from the weighted average of the survival rate for the seedlings in the corresponding F_2:3_ families.

### DNA extraction, map construction, and QTL analysis

Total genomic DNA was extracted from fresh leaves of individual plants using the CTAB method. PCR products were separated on a 6% denaturing or non-denaturing polyacrylamide gel and detected by silver staining. Genomic sequence, SSR, and InDel markers were obtained from GRAMENE (https://archive.gramene.org/markers/microsat/).

The bulked segregant analysis (BSA) method was applied to detect tightly linked makers of tolerance genes/QTLs. It referred to the method previously described by Qiu et al. [26]. The JoinMap 3.0 [36] was used to construct the region of interested genetic linkage map of markers; and the MapQTL 5 [35] was applied to perform the QTL analysis of the BPH resistance. The same method was performed to identify the resistance gene *Bph1* on chromosome 12 directly according to the results described by Alam and Cohen [1].

### Host plant tolerance to the BPH

To characterize the tolerance of plants with resistance genes/QTLs to BPH, changes in the dry weight of individuals were determined before and after the plants were treated with BPH at the seedling and adult stages. The tests were conducted as described by Qiu et al. [29]. The seedlings were grown in individual 0.4 L plastic cups under natural conditions. One-week before treatment with BPHs, the plants were cultured in a greenhouse at a constant temperature (26–30 °C). Each plant (28-days-old) was treated with 15 s-instar nymphs, with the exception of the control plants. Four-days after the treatment, the plants were removed from the pots with the roots, cleaned, dried at 70 °C for 48 h, and then weighed individually. The experiments were performed with eight replicates.

To measure the tolerance of adult plants (50-days-old) to BPH infestation, we planted four seedlings at the three-leaf stage individually in one plastic bucket (diameter 29 cm, height 25 cm). Only one main stem and one tiller were remained for each plant before 7 days prior to infestation. Then each bucket was treated with 80 s-instar nymphs and enclosed in a fine and light transmitting mesh. Fourteen-days after the treatment, the plants were treated as the seedlings. A total of eight buckets were surveyed for both the treatment and control. The tolerance index (TI) calculated following the formula described by Cohen et al. [8], as follows: TI = *W*_*t*_/*W*_*c*_ × 100; where, *W*_*t*_ and *W*_*c*_ are the dry weight of the infested and control plants, respectively.

### BPH performance and development on rice plant

The host choice test was performed as described by Qiu et al. [26]. Three 14-day-old seedlings of pre-NILs with one or two genes and KWQZ were transplanted in a plastic bucket (15 cm diameter, 14 cm height) with seedlings forming a triangle. The bucket was then completely covered with fine and light transmitting mesh; eight buckets were surveyed. To investigate the host choice of the BPH, 60 s–third instar nymphs were placed in each bucket and allowed to choose a host plant (35-days-old) on which to feed and reproduce over a 120 h period. The BPH insects settled on each plant were counted at 3, 6, 24, 48, 72, 96, and 120 h after release.

To quantify the excretion of BPH honeydew and the increase in growth weight, the seedlings were treated the same as described for the host selection test. One pre-weighed BPH insect with a short wing was released in a rectangle parafilm bag (3.5 cm length, 3 cm width), which was also pre-weighed and fastened on the rice shoot. Each BPH was collected and the weight was recorded after 2 days; the bag containing honeydew excretion was also weighed. Each plant had two parafilm bags, and eight plants for each genotype.

### Statistical analysis

Statistical analysis of the data was performed with SPSS 13.0 (SPSS Institute Inc., Chicago, IL, USA). The resistance data were analyzed by one-way ANOVA and comparing the LSD tests at a 5% significance level.

## Results

### BPH resistance and tolerance evaluation and genetic analysis

**Fig. 1:**
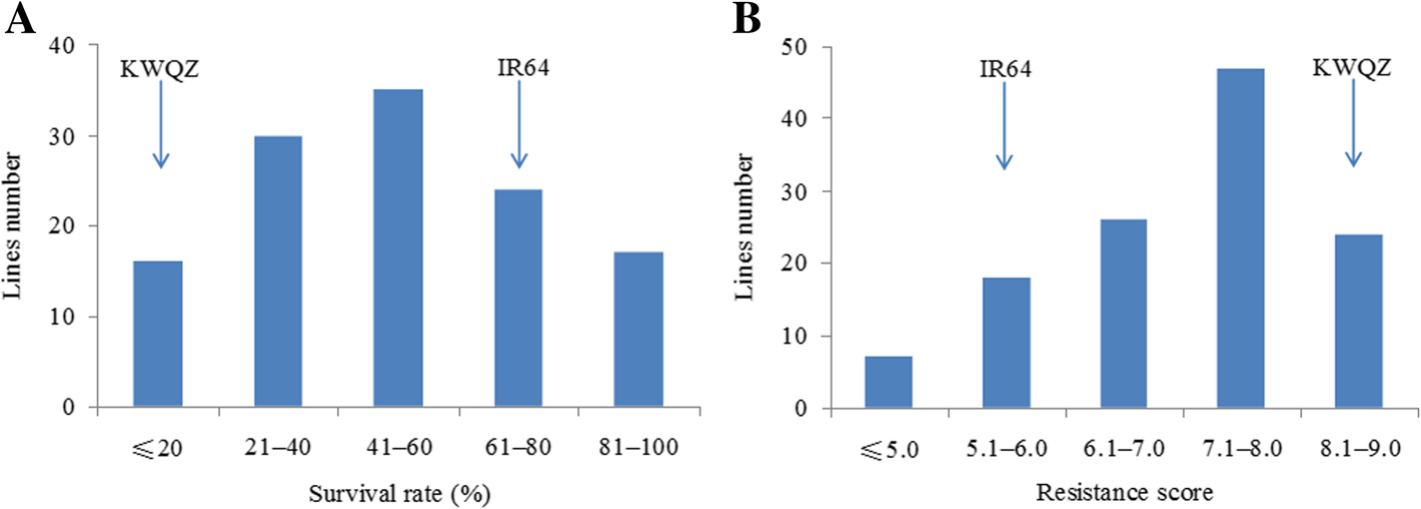
Frequency distribution of the BPH resistance evaluation of F_2:3_ lines derived from the cross KWQZ/IR64. **a**, Seedlings survival rate. Three-week-old seedlings were treated with five to six BPHs per plant for 20 days. The survival rates of the parents IR64 and KWQZ were 67 and 18%, respectively. A lower survival rate indicates more susceptible. **b**, BPH resistance scores. Three-leaf seedlings were treated with eight BPHs per plant for 9–10 days. The average resistance scores of the parents IR64 and KWQZ were 5.6 and 8.7, respectively. A lower score indicates higher resistance

Although almost 20 years passed, we still found that the rice variety IR64 presents moderate resistance to the BPH insects collected from the rice field of Nanning, Guangxi. The average resistance score is 5.6 according to the criterion described by International Rice Research Institute (IRRI) in the seedling bulk test. Moreover, the IR64 seedlings had a relative high survival rate compared with KWQZ when treated with BPH; the average scores were 67 and 18%, respectively (Fig. 1a, F = 11.2, *P* = 0.004). This suggested that IR64 showed resistance and tolerance to BPH.

To further analyze the resistance mechanism of IR64, we mapped the resistance genes/QTLs based on the seedling bulk test and survival rate detection, respectively, in the greenhouse. A previous study showed that a tolerance QTL on chromosome 1 explained 5.1 and 7.1% of phenotypic variation, respectively, in a doubled-haploid mapping population infested with two different BPH populations [1]. We also observed the tolerance effect in our recent insect resistance test with the analysis of seedling survival rate. Therefore, we considered the tolerance QTL to be a major gene and a BSA method was used to assay a F_2:3_ mapping population. Thus, measuring the tolerance phenotype showed that the survival rate ranged from 0 to 82%, and most ranged from 21 to 60% in the F_2_ population. Several lines exhibited an extreme phenotype compared with the parents (Fig. 1a). If a survival rate of ≤40% was considered non-tolerance, 46 and 76 individuals were non-tolerance and tolerance, respectively, in the mapping population.

The average resistance scores of F_2_ lines varied from 4.6 to 9.0 based on the seedling resistance test, and most of them were within the range 6.1–9.0. Notably, 51 resistance plants and 71 susceptible plants were detected if a resistance score between 7.1 and 9.0 was considered to be susceptible (Fig. 1b). The result suggested that most of F_2_ lines showed susceptible to the BPH in the seedling bulk test.

### Tolerance QTL mapping

**Fig. 2:**
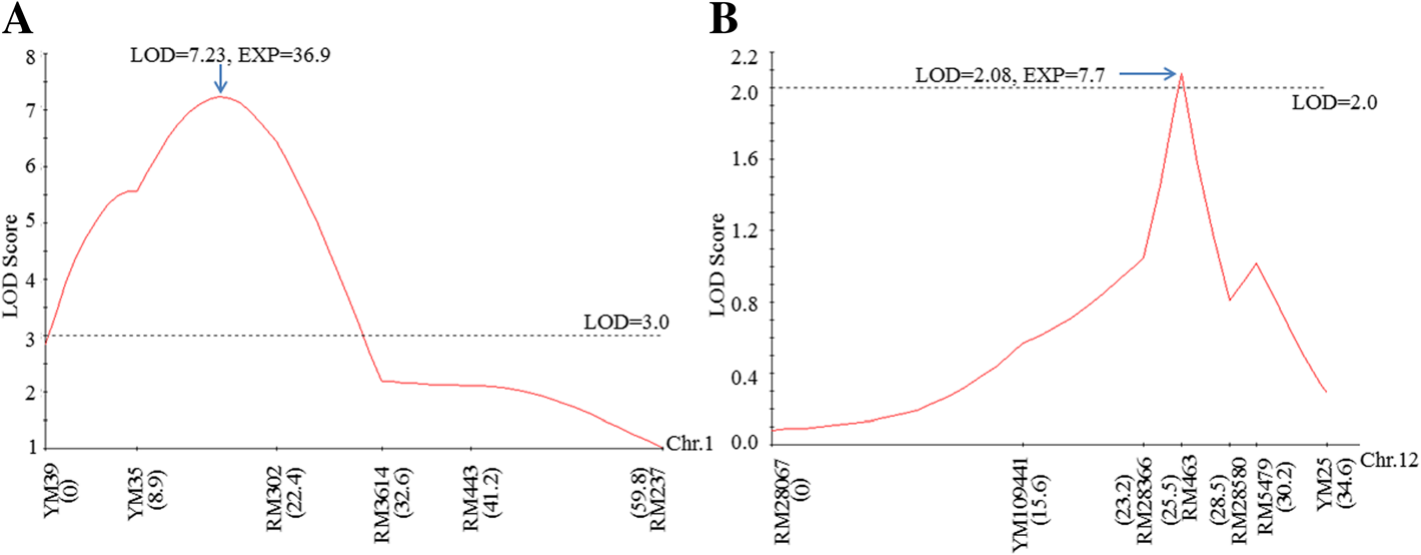
Location of the resistance genes on the linkage map of rice chromosomes constructed using an F_2_ population derived from KWQZ/IR64. **a**, *Bph37* on the chromosome 1. **b**, *Bph1* on chromosome 12. Markers are presented along the X-axis with distances (in cM) as shown. LOD scores are presented on the Y-axis. EXP, phenotypic variance explained by the locus

To identify the tolerance QTL/gene, a BSA method was performed to screen the tightly linked markers. In total, 960 rice molecular markers were applied to detect the DNA bulks. Subsequently, two polymorphic markers, RM302 and YM35, from the same region of chromosome 1 were detected between the two bulks, which suggested that a tolerance resistance gene was located in this region. Then, several polymorphic markers between the parents were applied to detect the genotype of F_2_ individuals and a local genetic linkage was constructed based on the selected genotype with JoinMap 3.0 (Fig. 2a). Next, an interval QTL mapping on the target chromosome region was performed using MapQTL 5. Subsequently, one locus with the largest LOD score of 7.23 was found between the markers RM302 and YM35. The locus explained 36.9% of phenotypic variation in the mapping population (Tables 1 and 2). In addition, the closest markers RM302 and YM35 also had a large LOD score of 6.43 and 5.57 and explained 28.6 and 25.3% phenotypic variation, respectively. As the detected QTL confers tolerance to the BPH in the population, it was tentatively designated as *Bph37* according to McCouch and CGSNL [21].

**Table 1:** Chromosomal locations and biometrical characteristics of genes for brown planthopper resistance

Trait	Peak interval		Chromosome	Peak LOD	% Variance explained	Additive
Tolerance	YM35	RM302	1	7.23	36.9	−4.68
Antibiosis	RM28366	RM463	12	2.08	7.7	−0.65

The genetic effect estimated using progeny data with MapQTL 5. An additive effect was equal to half the difference in the trait value between two homozygotes. % Variance explained is the percentage of total phenotypic variance explained by the locus

**Table 2:** Polymorphic markers associated with BPH resistance genes

Marker	Trait	Forward primer (5'–3')	Reverse primer (5'–3')	Product size (bp)	Type
YM35	Tolerance	GCATGCTGTATTACAATTACGA	GACAACGTACCACAGATTCC	160	InDel
RM302	Tolerance	TGCAGGTAGAAACTTGAAGC	AGTGGATGTTAGGTGTAACAGG	251	SSR
RM28366	Antibiosis	AGGATACTTCGAAAGACTGAGC	GTTTGTCACGAGAGCTTCTACC	454	SSR
RM463	Antibiosis	GAGGATTAATTAGCGTGTGACC	GTCGTGACATCTACTCAAATGG	388	SSR

### BPH resistance gene mapping

Previous studies have shown that the rice variety IR64 carries the major resistance gene *Bph1* and several other minor resistance QTLs [1, 33]. To detect the resistance effect conferred by *Bph1* in the same population, we surveyed the resistance score of F_2:3_ lines and mapped it again. According to the chromosome location of *Bph1*, seven polymorphic markers between the parents were used to analyze the genotype of F_2_ individuals. Then, a local genetic linkage map was constructed, and the gene was evaluated with interval QTL mapping method. The way was identical to the tolerance gene identification. As a result, one locus with the largest LOD score of 2.08 was detected between the markers RM28366 and RM463, which explained 7.7% of phenotypic variance in the mapping population (Fig. 2b; Tables 1 and 2). The locus was basically identical to the location of *Bph1* [1, 17]. It suggested that the resistance gene *Bph1* contributes less in the resistance of IR64.

### Evaluation of BPH resistance of pre-NIL

**Fig. 3:**
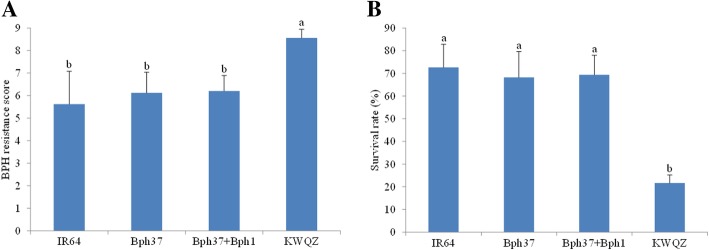
BPH resistance phenotype in pre-NIL with one or two genes and the parents (IR64 and KWQZ) measured by using the seedling bulk test. **a**. resistance scores, **b**. survival rates. Each bar represents the mean of nine replicates, *error bars*, SD. Different lowercase letters above bars indicate that the means (*bar*) are significantly different at *P =* 0.05. The survival rate was equal to the surviving plants divided by total plants of each line

As indicated in the seedling bulk test, the rice variety IR64 and pre-NIL with one or two resistance genes *Bph1* or *Bph37* showed moderate resistance to the BPH insects. The average resistance scores were 5.62, 6.12, 6.21, and 8.56 for IR64, pre-NIL-*Bph37*, pre-NIL-*Bph37* + *Bph1*, and KWQZ, respectively (Fig. 3a). There was no statistical difference among the resistance parent and lines with one or two resistance genes (*P* > 0.05). However, a significant difference in seedling survival rate was observed between the susceptible parent (21.6%) and the pre-NILs with resistance genes (68.2% for pre-NIL-*Bph37*, 69.4% for pre-NIL-*Bph37* + *Bph1*) (F = 10.6, *P* ≤ 0.01 for pre-NIL-*Bph37* and KWQZ; F = 12.2, P ≤ 0.01 for pre-NIL-*Bph37* + *Bph1* and KWQZ; Fig. 3b).

### Tolerance evaluation of pre-NIL after BPH infestation

**Fig. 4:**
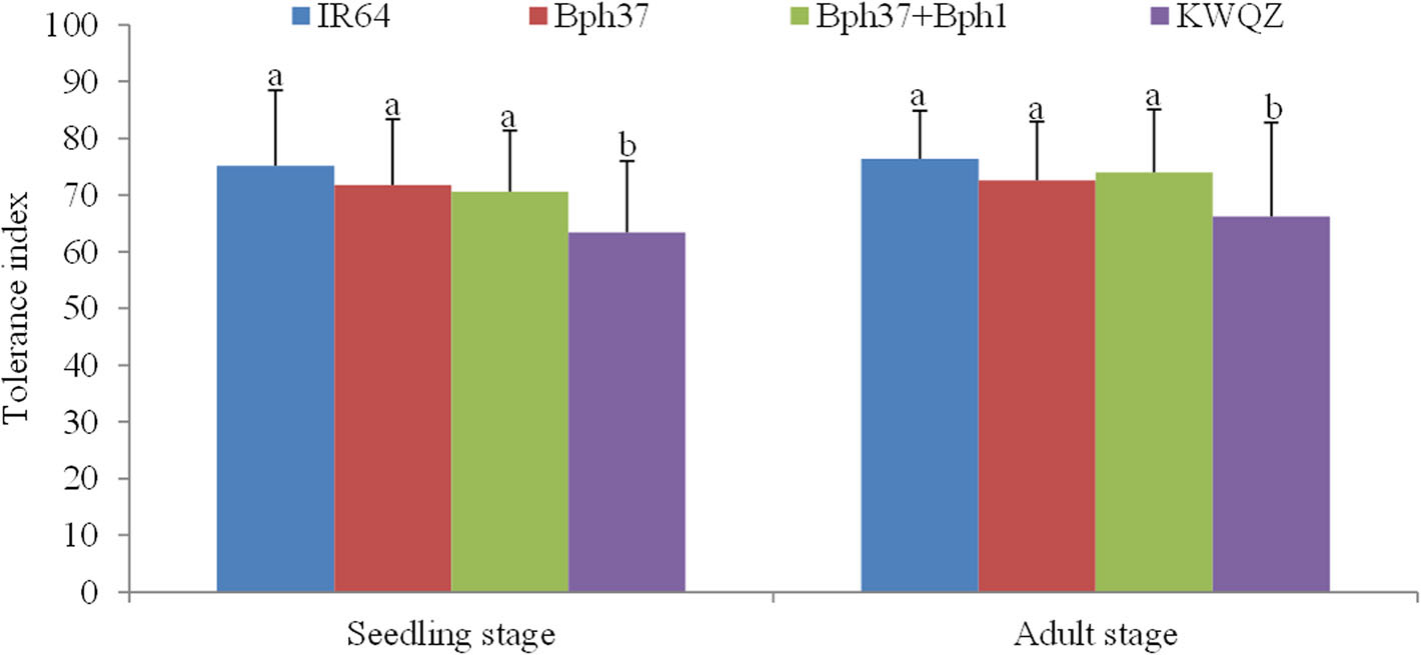
Plant tolerance of different genotypes to the BPH insects. Bars represent means of eight replicates. *Error bars* represent the SD. Different lowercase letters above bars indicate that the means (*bar*) are significantly different at *P =* 0.05. Tolerance effect was evaluated with TI = *W*_*t*_/*W*_*c*_ × 100, *W*_*t*_ and *W*_*c*_ are the dry weight of the infested and control plants, respectively

Changes in the plant dry weight of pre-NIL and KWQZ seedlings or adult plants were used to characterize the tolerance conferred by the associate gene. As a result, the dry weight of plants infested with BPH was decreased comparing with the untreated plants, especially for the susceptible plants (Fig. 4). By the fourth day after BPH infestation, the dry weight of IR64, pre-NIL-*Bph37*, and pre-NIL-*Bph37* + *Bph1* reduced by 24.8, 28.2, and 29.4%, respectively, while the change of KWQZ reduced significantly by 36.6% (F = 6.7, *P* = 0.01 for IR64 and KWQZ; F = 5.2, *P* = 0.03 for pre-NIL-*Bph37* and KWQZ; F = 4.6, *P* = 0.04 for pre-NIL-*Bph37* + *Bph1* and KWQZ). The same trend was observed when the tillering plants treated with insects. The plant dry weight of IR64, pre-NIL-*Bph37*, pre-NIL-*Bph37* + *Bph1*, and KWQZ plants decreased by 23.6, 27.4, 26.0, and 33.8%, respectively, after BPH feeding for 15 days (F = 7.2, P = 0.01 for IR64 and KWQZ; F = 4.9, P = 0.04 for pre-NIL-*Bph37* and KWQZ; F = 5.4, *P* = 0.02 for pre-NIL-*Bph37* + *Bph1* and KWQZ). The findings suggest that the gene *Bph37* plays an important role in the resistance of IR64 via tolerance to BPH.

### Antixenotic effect on the BPH insects

**Fig. 5:**
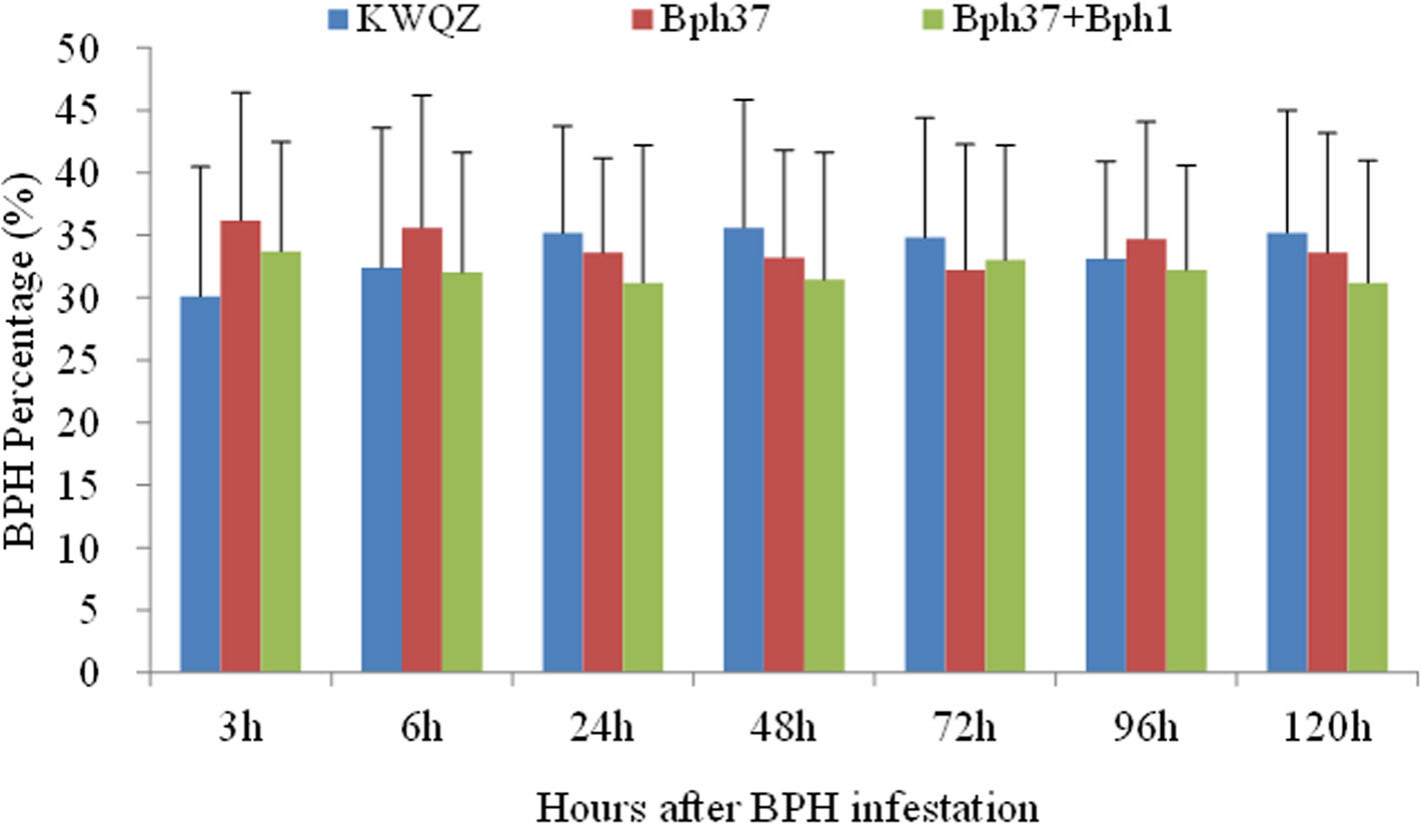
BPH host choice among the different genotype plants. *Bars* represent means of eight replicates. *Error bars* represent the SD

The BPH host choice test was performed among plants with different genotypes. More BPH insects settled on the plants with *Bph37* at 3 h (36.2, 33.7, 30.1 for pre-NIL-*Bph37*, pre-NIL-*Bph37* + *Bph1* and KWQZ, respectively) and 6 h (35.6, 32, 32.4 for pre-NIL-*Bph37*, pre-NIL-*Bph37* + *Bph1* and KWQZ, respectively) comparing with that on the susceptible plants. But more BPHs then attached to the shoots of KWQZ during the period of 24–120 h after release (Fig. 5). Generally, BPH showed no obvious host preference among different types of plants according to the observing insect numbers. And one-way ANOVA analysis also showed no significant difference in BPH preference among the plants with different genes during the period of 120-h infestation. This result indicates that antixenotic factors were not presented in the resistant plants with *Bph37* or *Bph37* + *Bph1*.

### BPH performance on the host plants

**Fig. 6:**
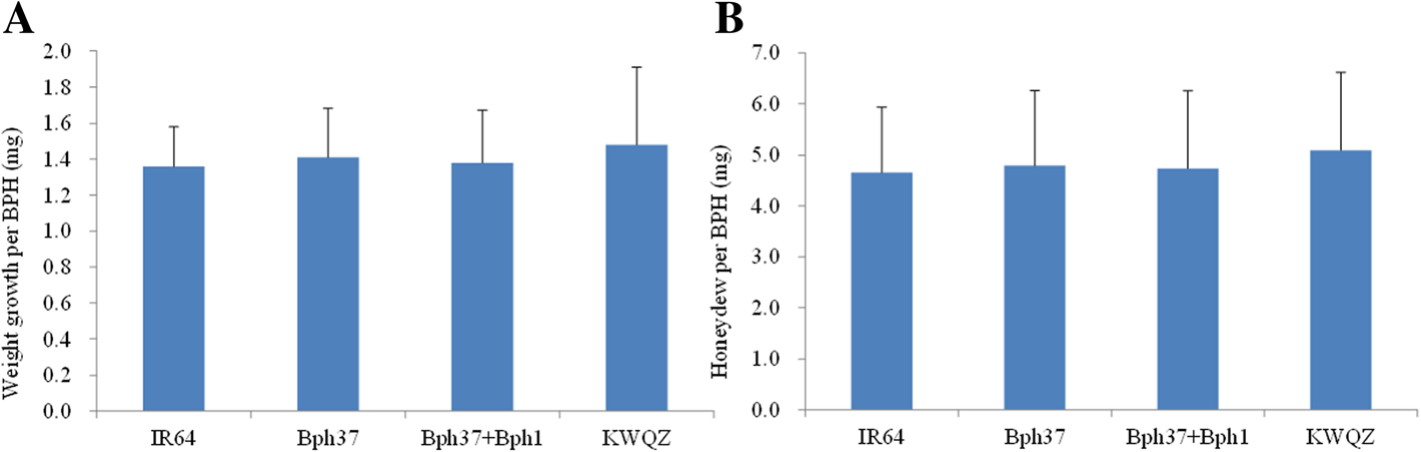
BPH development on plants of different genotypes. **a**. Increase in BPH growth weight on resistant and susceptible plants, **b**. BPH honeydew excretion on resistant and susceptible plants. *Bars* represent means of eight replicates. *Error bars* represent the SD

The increase in BPH growth weight and quantity of honeydew excretion on plants with different genotypes were compared to determine whether the resistance genes affected BPH growth and development. The BPH weight gain was 1.36, 1.41, 1.38, and 1.48 mg on the IR64, pre-NIL-*Bph37*, pre-NIL-*Bph37* + *Bph1*, and KWQZ plants, respectively; and it reduced 8.1, 4.7, and 6.8%, respectively, comparing with the susceptible plants. As for the honeydew excretion, it reduced 8.8% (4.65/5.1), 6.1% (4.79/5.1), and 6.1% (4.74/5.1) on the IR64, pre-NIL-*Bph37*, and pre-NIL-*Bph37* + *Bph1*, respectively, compared to that on the KWQZ (Fig. 6a). It must be noted that less weight gain or honeydew excretion were measured on the resistance plants comparing with the susceptible plants. However, no significant differences were detected among the plants with different genotypes (*P* > 0.05; Fig. 6b). This result indicates that the BPH development was not significantly inhibited on the resistance plants.

## Discussion

Plants would present antibiosis, antixenosis, or tolerance when attacked by insects [18]. Antibiosis is the most common reaction induced by the plant and insect interaction. For instance, the rice lines carrying *Bph6*, *Bph9*, or *Bph14* exhibited significant antibiosis to the BPH insects [11, 26, 40]. This may be because it can produce harmful secondary metabolites, which can slow or suppress insect development or growth. Bird et al. [4] indicated that opium poppy can produce a diverse array of pharmaceutical alkaloids, which restrict insect growth. Antixenosis refers to plants that can avoid pest-related damage. Qiu et al. [28] detected one antixenotic QTL, *Qbph8*(*t*), from resistance variety Swarnalata which conferred a host preference behavior.. Tolerance refers to plants being able to sustain tissue loss with little or no decrease in fitness relative to that in the undamaged state [24, 32]. For example, two wild relatives of maize have a greater number of tillers than the domesticated species and are more tolerant of stem borer damage [31]. In the present study, with detection of seedling survival rate, we mapped a BPH resistance gene which was tolerance to BPH on chromosome 1 between markers RM302 and YM35; the LOD score was 7.23 and explained 36.9% phenotypic variation in the population. Previous studies have reported that one QTL confers tolerance at the same chromosomal region in IR64 [1, 33]. Here, we mapped it as a major QTL with analysis of seedling survival rate and different mapping population. And we then characterized it with pre-NIL through host choice test, weight gain, and honeydew excretion. The results would be beneficial for understanding of *Bph37*. Finally, the tightly linked SSR markers could be applied to marker-assisted selection. In all, the present research provided a starting to advance the study of *Bph37* which resistance to BPH via tolerance. It’s possible a suitable choice to control the rice pest.

The evolution of BPH biotypes destroys host-plant resistance when reared on the plants with a single resistance gene. To address this, scientists commonly pyramid major resistance genes, such as *Bph14 + Bph15, Bph12 + Bph6,* and *Bph25 + Bph26* into cultivars by molecular markers to increase the level of resistance [20, 22, 27]. However, it remains unknown whether the cultivars have more durable resistance. The study of IR64 clearly indicated that pyramiding a major resistance gene and minor resistance genes/QTLs could effectively prolong the resistance trait [1, 2, 8]. Here, we showed that resistance conferred by *Bph37* was very important for resistance to BPH. Therefore, the detected gene and tightly linked molecular markers may be effectively used in practical breeding programs, and the mechanism of resistance should be elucidated in future studies.

Resistance traits in plants, especially those conferred by antibiotic genes, confer heavy selection pressure on herbivore traits, with the exception of tolerance [30]. The plants carrying *Bph37* exhibited moderate resistance to BPH, which would impose a relatively moderate selection pressure on the insects and does not favor the evolution of BPH populations [8]. On the contrary, it should be beneficial for controlling BPH outbreaks. Previous studies have indicated that rice cultivars containing major and minor genes/QTLs have a stronger antixenosis compared to plants containing a single gene/QTL [28]. Furthermore, it also confers more durable resistance to the BPH insects [5, 39]. Thus, *Bph37* is an important and preferential gene for resistance gene pyramiding. Moreover, most identified BPH resistance genes are located on chromosomes 4, 6, and 12 [6], whereas *Bph37* is located on chromosome 1. MAS with tightly linked markers RM302 and YM35 is easy to perform, with no consideration for gene linkage drag needed. Therefore, this should be beneficial for the development of BPH-resistant rice varieties.

## Conclusions

One major BPH resistance gene *Bph37* was successfully mapped to a chromosomal region harbored by markers RM302 and YM35 on chromosome 1. Moreover, the reported gene *Bph1* was detected to be conferred minor resistance effect when evaluated the single gene lines with *Bph37* or pyramided lines with *Bph37* and *Bph1*. Interesting, the lines carrying *Bph37* mainly confers tolerance to the BPH insects which should be beneficial for understanding of the resistance mechanism of BPH resistance to rice and for insect-resistance rice breeding programs.

## Data Availability

Not applicable.

## Abbreviations

ANOVA: Analysis of variance
BPH: Brown planthopper
BSA: Bulked segregant analysis
CTAB: Cetyltrimethylammonium bromide
InDel: Insertion/deletion
LSD: Least significant difference
PCR: Polymerase chain reaction
pre-NILs: Preliminary-near-isogenic-lines
QTL: Quantitative trait locus
SSR: Simple sequence repeat
